# *PIK3AP1* and *SPON2* Genes Are Differentially Methylated in Patients With Periodic Fever, Aphthous Stomatitis, Pharyngitis, and Adenitis (PFAPA) Syndrome

**DOI:** 10.3389/fimmu.2020.01322

**Published:** 2020-07-23

**Authors:** Ema Lovšin, Jernej Kovač, Tine Tesovnik, Nataša Toplak, Daša Perko, Tomaž Rozmarič, Maruša Debeljak, Tadej Avčin

**Affiliations:** ^1^Department of Allergology, Rheumatology and Clinical Immunology, University Medical Centre Ljubljana, University Children's Hospital, Ljubljana, Slovenia; ^2^Faculty of Medicine, University of Ljubljana, Ljubljana, Slovenia; ^3^Department for Special Laboratory Diagnostics, University Medical Centre Ljubljana, University Children's Hospital, Ljubljana, Slovenia; ^4^Department of Pediatrics and Adolescent Medicine, Medical University of Vienna, Vienna, Austria

**Keywords:** PFAPA, differential methylation, PIK3AP1, SPON2, MSRE-qPCR, MeDIP, MBD

## Abstract

Periodic fever, aphthous stomatitis, pharyngitis, and adenitis (PFAPA) syndrome is the most common autoinflammatory disease in children and is often grouped together with hereditary periodic fever syndromes, although its cause and hereditary nature remain unexplained. We investigated whether differential DNA methylation was present in DNA from peripheral blood mononuclear cells (PBMC) in patients with PFAPA vs. healthy controls. A whole-epigenome analysis (MeDIP and MBD) was performed using pooled DNA libraries enriched for methylated genomic regions and identified candidate genes, two of which were further evaluated with methylation-specific restriction enzymes coupled with qPCR (MSRE-qPCR). The analysis showed that the *PIK3AP1* and *SPON2* gene regions are differentially methylated in patients with PFAPA. MSRE-qPCR proved to be a quick, reliable, and cost-effective method of confirming results from MeDIP and MBD. Our findings indicate that a B-cell adapter protein (*PIK3AP1*), as the PI3K binding inhibitor of inflammation, and spondin-2 (*SPON2*), as a pattern recognition molecule and integrin ligand, could play a role in the etiology of PFAPA. Their role and the impact of changed DNA methylation in PFAPA etiology and autoinflammation need further investigation.

## Introduction

Periodic fever syndrome with adenitis, pharyngitis, and aphthous stomatitis (PFAPA) belongs to the group of autoinflammatory diseases (AID). This group of disorders is marked by increased inflammation associated with the innate immune system, and most of the disorders are inherited in a Mendelian pattern (1, 2). Of the various autoinflammatory diseases, many now have a confirmed known genetic cause. However, PFAPA syndrome still has an unknown genetic background and pathogenesis (3).

PFAPA was first described by Marshall et al. (4). Its most common feature is periodic fever, while its other features are more variable: pharyngitis, aphthous stomatitis, and cervical adenopathies (5, 6). Episodes have an early onset, usually in patients around 2–3 years of age, mainly before the age of five. Episodes can reoccur for several years, followed by disease remission, and leave no long-term health consequences (5–7). The syndrome is considered sporadic and has been described as a non-inherited syndrome; however, familial cases of PFAPA have been reported, suggesting a potential genetic origin (3, 8–11). PFAPA could be caused by cytokine dysregulation linked to genetic variants of autoinflammatory disease-associated genes (12). Studies also point to altered complement activation and IL-1 production in PFAPA patients (13), as well as to IL-1β dysregulation (14). Since PFAPA shares clinical similarities with monogenic fever syndromes, several studies have investigated the possible involvement of the genes responsible for Familial Mediterranean Fever (*MEFV* gene), TNF-Receptor Associated Periodic Syndrome (*TNFRSF1A* gene), Mevalonate kinase deficiency (*MVK* gene), and Cryopyrin-Associated Periodic Syndrome (*NLRP3* gene) in PFAPA cohorts (15). A variant in *CARD8* has been associated with more severe cases of PFAPA. CARD8 acts as a negative regulator of the inflammasome; its decreased ability to bind NLRP3 could partly contribute to exacerbated inflammasome responses, although probably not on its own (16). Genes known to be involved in inflammation or in autoinflammatory disorders seem to contribute to a predisposition to PFAPA syndrome, suggesting complex genetic inheritance and interaction with non-genetic factors (12, 14, 17–20).

Several inflammasome-related genes have been found to have increased expression when demethylated during the differentiation of monocytes to macrophages, specifically *AIM2, NLRC5, PYCARD, CASP1*, and *PSTPIP2*, and their targets *IL1A, IL1B*, and *IL1RN*. Furthermore, untreated patients with Cryopyrin-Associated Periodic Syndrome (CAPS) have exacerbated DNA methylation-dependent regulation of the inflammasome product genes *IL1B, ILR1N, NLRC5*, and *PYCARD*, while patients with CAPS undergoing anti-IL-1β treatment have displayed demethylation levels in stimulated monocytes similar to those seen in healthy subjects (21). Different methylation levels of the *MEFV* gene have been observed in Familial Mediterranean Fever patients compared to healthy controls (22). Additionally, DNA methylation plays an important role during hematopoietic differentiation to a myeloid vs. a lymphoid lineage (23).

Since studies suggest that DNA methylation plays a role in other autoinflammatory diseases, we hypothesized that specific methylation patterns may be aberrant in at least a small portion (population) of peripheral blood mononuclear cells (PBMC) in PFAPA patients. To identify the potentially relatively small change in the methylation patterns due to the specifics of a PBMC-derived DNA sample and to confirm whether the PFAPA cohort differs in DNA methylation patterns from healthy controls, a whole-epigenome analysis was performed using pooled DNA libraries enriched for methylated genomic regions using Methylated DNA Immunoprecipitation (MeDIP) and Methyl-CpG-binding domain (MBD). We identified several candidate genes with differential methylation, two of which (*PIK3AP1* and *SPON2*) were chosen based on their involvement in the inflammation pathways and were further evaluated with MSRE-qPCR. To our knowledge, no research had previously been performed regarding differentially methylated DNA in PFAPA patients.

## Materials and Methods

### Participants

Clinical data and samples of 75 patients (44 boys and 31 girls) with PFAPA syndrome at the University Children's Hospital Ljubljana were collected from 2008 to 2016. The median age of the patients when blood was taken for the DNA analysis was 4.1 [Interquartile range, IQR 3–5.8] years. Patients were not in an active state of the disease when samples for DNA isolation were taken. Prior to donating blood for DNA analysis, 15 (20%) had received methylprednisolone in the past but not in the month prior to blood donation (except for one individual, who had received treatment 2 weeks prior). Samples from 65 apparently healthy children (35 boys and 30 girls) of Slovenian ethnicity whose median age was 5.3 [IQR 5.2–5.4] were included in the study as healthy controls. The parents of each child included in the study were informed about the aim of the study and signed a written informed consent form for inclusion in the study. The study was approved by the Ethics Committee of the Republic of Slovenia and was conducted according to the principles of the Helsinki Declaration.

### Samples

Five ml of peripheral blood was taken for DNA isolation. Blood was taken from patients during routine venipuncture at follow-up visits. Blood of the healthy controls was taken during routine health examinations of the children. DNA isolation was performed using the FlexiGene isolation kit (Qiagen, Germany) according to the recommended protocol. The DNA was stored at 4°C.

### MeDIP and MBD Data Analysis

For each sample pool (PFAPA vs. healthy), three separate NGS libraries were generated for each enrichment method (MBD or MeDIP). All 36 libraries were combined and simultaneously sequenced on the MiSeq Illumina sequencer [MiSeq Reagent Kit v3 (150-cycle)] by a 2 × 75 paired-end run. Sequencing was repeated until each separate library had acquired at least 10 million pair-end reads. The acquired dataset was filtered for low-quality reads and duplicates aligned by the BWA-MEM aligner (24) and followed by MACS2 (25) analysis of narrow and broad peak regions. The callable regions identified by the MACS2 algorithm were used to count reads in each peak region for each library by the featureCounts algorithm (26). Using DeSeq2 (27), the algorithm peaks were compared to identify differentially methylated regions via differences in normalized read counts. The Benjamini-Hochberg procedure, which controls false discovery rate (FDR), was used to identify true differentially methylated regions (DMRs). DMRs were annotated for the overlapping or proximal gene regions. DMRs associated with the immune system were further verified using MSRE-qPCR.

### Primer Selection and Evaluation, MSRE-qPCR Verification of DMRs

PCR primers were designed using the Primer 3 online tool (http://primer3.ut.ee/) and SNP Check (https://genetools.org/SNPCheck/snpcheck.htm) according to the established laboratory protocol, covering the whole sequence identified by MBD or MeDIP. Since the identified sequences were longer than is optimal for qPCR amplicons, primers were chosen in pairs to cover the first and second halves of the sequence (Table 1). The primers were first evaluated with PCR and 2% agarose gel electrophoresis (SYBR staining) and with a 50 bp DNA ladder (N0556S, New England Biolabs), figure of Electrophoresis gel is available in Supplementary Table 2. PCR was performed with Go Taq G2 Green Master Mix according to the protocol.

**Table 1:** Primer sequences and PCR product length, genomic position and location within the gene.

**Gene**	**Accession number**	**Primer**	**Sequence**	**Product size [bp]**	**GRCh37 position**	**Location**
HBB	NM_000518	HBB F	GGATGAAGTTGGTGGTGAGG	231	11:5247959–5248189	Exon 1–2
		HBB R	CAGCATCAGGAGTGGACAGA			
PIK3AP1	NM_152309	PIK_1 F	AAAAGAGTTAAATAGGCCGGGCG	120	10:98425881–98426000	Intron 2
		PIK_1 R	GTTTCACCATGTTAGCCAGGATG			
		PIK_2 F	GATCACAAGGTCAGGAGATCGAGA	238	10:98425953–98426190	Intron 2
		PIK_2 R	TTTGTTTGTTTGTTTGAGATGGAGTC			
SPON2	NM_012445	SPON_1 F	TAATTACTGCTGCTCCTCAAGACG	174	4:1163329–1163502	Intron 5
		SPON_1 R	GGACTTCAGACTTTCCCGAGGA			
		SPON_2 F	CTCCTCGGGAAAGTCTGAAGTC	248	4:1163480–1163727	Intron 5
		SPON_2 R	CATTCTCCTAGCTCTTCCAGGC			

After successful evaluation for specificity and annealing temperature, evaluation of the qPCR assay was performed. We used Luna Universal qPCR Master Mix (New England BioLabs) with a fast cycling profile. Standard curves were prepared for each primer pair to determine the efficiency (E) of the designed primers. Efficiency calculations were calculated online with the NEBioCalculator for qPCR Quantification (https://nebiocalculator.neb.com/#!/qPCRGen) (Table 2).

**Table 2:** qPCR reaction efficiencies.

**qPCR reaction**	**E [$10^{\frac{-1}{\text{slope}}}$]**	**Efficiency**	***R*^**2**^**	**Slope**
HBB	1.945	0.945	0.998	−3.46
PIK_1	2.231	1.231	0.984	−2.87
PIK_2	2.117	1.117	0.961	−3.07
SPON_1	1.906	0.906	0.996	−3.57
SPON_2	1.771	0.771	0.992	−4.03

### Enzyme Restriction

The regions selected for MSRE-qPCR had multiple CpGs between forward and reverse primer annealing sites. The selected enzymes (MspJI and McrBC; Tables 3, 4) cover almost all CpGs; however, for successful digestion and subsequent evaluation, at least one of the targeted CpGs must be methylated. Furthermore, the MspJI enzyme can also recognize methylated C in front of any base (Table 3). However, since, in humans, mostly CpGs are methylated, only CpGs were counted for the number of candidate restriction sites (Table 4). Beta globin amplicon, which does not contain any CpGs, was used as a reference. Equal amounts of DNA were used in control and restriction reaction. First, 200 ng of DNA was digested in accordance with the manufacturer's protocol by 2 units of MspJI and 2 units of McrBC (both New England BioLabs) in a 25-μl reaction with CutSmart buffer for 1 h at 37°C, and this was followed by heat inactivation for 20 min at 65°C. Samples from the restriction reaction were then purified using Sample Purification Beads from a TruSight® One Sequencing Panel Kit according to the manufacturer's protocol.

**Table 3:** Recognition sites for methylation-sensitive enzymes (both New England Biolabs) used for MSRE-qPCR.

**Enzyme**	**Recognition site**
MspJI (R0661)	^m^CNNR
McrBC (M0272)	Pu^m^CG

m *C represents 5-methylcytosine or 5-hydroxymethylcytosine, N represents any of the nucleotides, and R and Pu represent purines A or G*.

**Table 4:** Amplicon CG content, number of CpGs, and candidate restriction sites within amplicons.

	**Length [bp]**	**CG content [%]**	**Number of CpGs**	**Candidate restriction sites**
HBB	231	51.1	0	0
PIK_1	120	55.0	5	5
PIK_2	238	53.4	10	9
SPON_1	174	62.1	6	6
SPON_2	248	63.3	8	6

### Quantitative PCR

The purified digested and undigested DNA samples were used for relative qPCR quantification. qPCR assays were run in triplicates in 96-well plates using Luna Universal qPCR Master Mix (SYBR). In addition, melt curves were performed. Five pairs of primers [two for PIK3AP1, two for SPON2, and one for normalization (HBB, beta globin)] were analyzed on digested and undigested DNA samples for relative quantification using the ΔΔC_T_ method (28). This method analyzes relative changes in methylation of the target gene to the reference gene, and it assumes uniform PCR amplification efficiency across all reactions. Due to varying efficiencies in qPCR assays, we used efficiency corrected calculation (Equations 1–3) (28) for each obtained CT to produce more accurate estimates in relative quantification.

(1) $$\begin{array}{l} Ratio=\frac{{E_{\text{target}}}^{\Delta CT\left(\text{target}\right)}}{{E_{\text{reference}}}^{\Delta CT\left(\text{reference}\right)}} \end{array}$$

(2) $$\begin{array}{l} \Delta CT\left(target\right)=CT\left(undigested,\;target\;gene\right)\\ \;-CT\left(digested,\;target\;gene\right) \end{array}$$

(3) $$\begin{array}{l} \Delta \text{CT}\left(\text{reference}\right)=\text{CT}\left(\text{undigested},\text{ reference gene}\right)\\ \;-\text{CT}\left(\text{digested},\text{ reference gene}\right) \end{array}$$

Calculations for the ΔΔC_T_ method were done in Excel. Statistical data analysis was performed in the GraphPad Prism 8 program. A Mann-Whitney test was performed; the data represent relative expression ratios obtained with Equation (1). *P* < 0.05 was considered statistically significant, and *p* < 0.01 was considered statistically highly significant.

## Results

### Clinical Characteristics of PFAPA Patients

All included participants fulfilled the clinical criteria for PFAPA syndrome (5). Included in our study were 44 (58.7%) boys and 31 (41.3%) girls. All patients were asymptomatic during the afebrile period. Pharyngitis (94.7%) and adenitis (89.3%) were the most present symptoms during febrile episodes, followed by abdominal pain (61.3%) and aphthous stomatitis (58.7%) (Table 5). Included in our cohort were three pairs of siblings (twin brothers, twin sisters, and two brothers). Positive family history, meaning that at least one first-degree relative had recurrent fevers or a tonsillectomy, was found in 73.4% of patients. Positive family history, either in the first or second degree, was present in 87.5% of patients.

**Table 5:** Demographic and clinical characteristics of PFAPA patients with family history and symptoms.

**Demographic and clinical characteristics of PFAPA patients**	
Total number of patients	75
Male	44 (58.7%)
Female	31 (41.3%)
Age at disease onset (mean ± SD)	2.1 ± 1.4 years
Age at giving a sample for DNA (mean ± SD)	4.5 ± 2.0 years
**Family history**	
Positive family history (first degree)	47/64 (73.4%)
Tonsillectomy in first-degree relative	32/65 (49.2%)
Tonsillectomy in first-degree relatives, more than one family affected	8/64 (12.5%)
Tonsillectomy in second-degree relative	19/64(29.7%)
Tonsillectomy in second-degree relative only, excluding those with tonsillectomy in first-degree relative	8/64 (12.5%)
Unknown	11/75 (14.7%)
**Symptoms**	
Pharyngitis	71 (94.7%)
Adenitis	67 (89.3%)
Abdominal pain	46 (61.3%)
Aphthous stomatitis	44 (58.7%)
Vomiting	28 (37.3%)
Joint pain	27 (36.0%)
Diarrhea	16 (21.3%)
Skin rash	9 (12.0%)

### Identification of DMRs With MDB and MeDIP

13.8 M (12.9–14.8 M) reads per library were collected on average, with 20% (17.2–22.8%) of the duplicates per library excluded from further analysis. The mean insert size per library was 318 bp (305–331 bp), and 91% (88.6–93.6%) of reads had proper pairs identified. MBD enrichment generated libraries with significantly higher %GC (63.3% [62.8–63.8%]) compared to MeDIP enrichment (49% [48.7–49.3%]). After cleanup of the datasets—by eliminating duplication, multiple alignment, and misalignment reads—the MACS2 algorithm identified 352,451 potential peaks in the MeDIP dataset and 100,902 peaks in the MBD dataset. To reduce the number of false-positive results in DMR analysis using DeSeq2, the FDR cutoff value was modified in such a way that the potential DMR set contained up to 1 (one) false-positive result. Consequently, the FDR cutoff value was set to *q* < 0.06 for the MeDIP dataset and *q* < 0.15 for the MBD dataset. After count tables were generated by the featureCount algorithm, the DeSeq2 identified 17 DMRs in the MeDIP dataset and seven DMRs in the MBD dataset. There was no overlap between the identified DMRs from the MeDIP and MBD datasets. All identified DMRs were annotated for proximal genes or other location-specific elements (intergenic space, regulatory elements, ncRNAs, etc.). The identified genomic elements/regions, listed in Supplementary Table 1, were analyzed for their potential role in (auto)immune response and consequently selected for further evaluation. Out of 24 identified DMRs, one DMR per enrichment method (MeDIP or MBD) with the most significant signal located near or within genes that could be reliably associated with the autoinflammation through published literature and public database search was further evaluated by MSRE-qPCR.

### Identification of DMRs With MSRE-qPCR

Of the differentially methylated sequences previously identified by using MDB and MeDIP, two regions of interest (ROIs) were chosen to perform our analysis based on the location and function of the gene. Of the top significant candidate genes, one was chosen from MeDIP (*PIK3AP1*, region 10:98425909–98426208, GRCh37) and one from the MBD method (*SPON2*, region 4:1163349–1163641, GRCh37). Relative quantification of digested and undigested DNA was performed with MSRE-qPCR in order to compare patients with healthy controls. The second intron region of *PIK3AP1* (10:98425909–98426208) was found to be more methylated in PFAPA patients by the MeDIP method and was verified by MSRE-qPCR, with a slight difference in the size of the differentially methylated region. MSRE-qPCR showed that only the second half (10:98425953–98426190) of the second intron region was over-methylated (*P* < 0.0001), while the first half (10:98425881–98426000) was not significantly different (*P* = 0.5079) compared to the healthy controls (Table 6).

**Table 6:** Descriptive statistics of each ROI with exact two-tailed *P*-values of the unpaired Mann-Whitney tests.

	**PIK_1 controls**	**PIK_1 patients**	**PIK_2 controls**	**PIK_2 patients**	**SPON_1 controls**	**SPON_1 patients**	**SPON_2 controls**	**SPON_2 patients**
*N*	64	74	64	75	59	65	61	69
Mean	0.1576	0.1892	0.03239	0.06402	0.2561	0.1912	0.2382	0.2911
Std. deviation	0.08185	0.1486	0.01146	0.09392	0.1584	0.1139	0.1505	0.1656
Std. error of mean	0.01023	0.01728	0.001433	0.01085	0.02063	0.01412	0.01927	0.01994
Lower 95% CI of mean	0.1371	0.1548	0.02953	0.04241	0.2148	0.163	0.1997	0.2514
Upper 95% CI of mean	0.178	0.2236	0.03526	0.08563	0.2974	0.2195	0.2768	0.3309
*P*-value	0.508		<0.0001		0.001		0.019	

Methylation results in the fifth intron region of the *SPON2* gene are conflicting. MBD defined the region of the fifth intron of *SPON2* (4:1163349–1163641) as more methylated in PFAPA patients, while MSRE-qPCR later showed that this is not the case for the whole region. The first half (4:1163329–1163502) of the fifth intron was, in fact, less (*P* = 0.001) methylated and the second half (4:1163480–1163727) was more (*P* = 0.0191) methylated in PFAPA patients compared to the healthy controls.

Results from both MeDIP and MBD were confirmed with MSRE-qPCR, with some differences. Firstly, MSRE-qPCR identifies smaller regions where differential methylation occurs, because qPCR reaction efficiency is limited with amplicon length. Secondly, because MSRE-qPCR has an amplicon size limit, it was able to reveal that the whole region of the fifth intron of the *SPON2* gene does not have higher methylation in PFAPA patients. Moreover, MSRE-qPCR requires only one methylated CpG at the ROI per single DNA molecule to detect a difference, as one cut site is enough to prevent PCR amplification of a particular DNA molecule.

**Figure 1:**
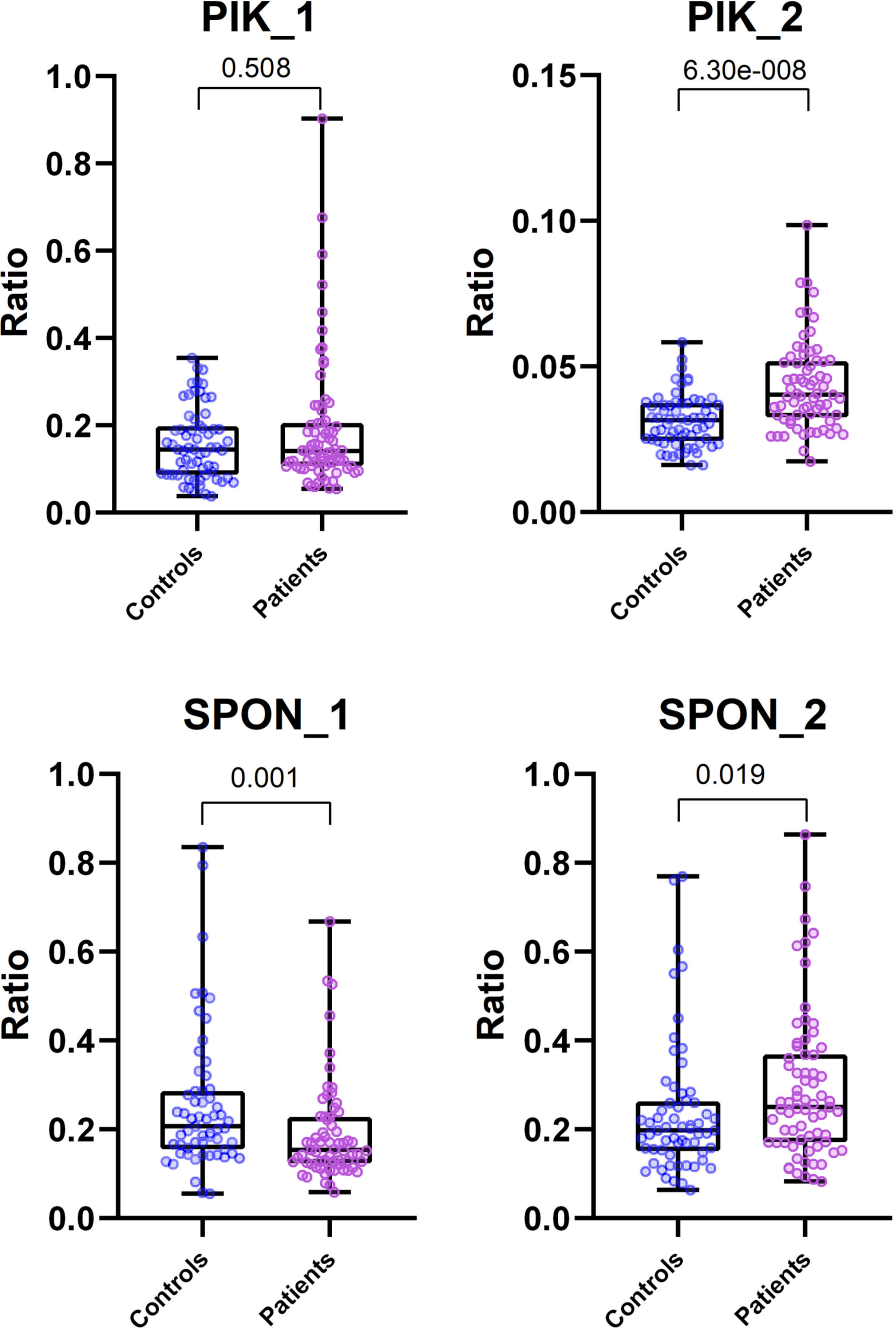
Comparison of MSRE-qPCR ratios between Control and Patient group for each ROI. Boxplots include scattered dots that represent relative expression ratio of Control (blue) or Patient (pink) sample. Also included at the top are exact two-tailed *P*-values of unpaired Mann-Whitney tests. All scatter plots are plotted with all data, except the plot for PIK_2 ROI, which was plotted with data without outliers (ROUT method, Q = 0.1%) for visualization purposes. Mann-Whitney tests for all ROIs were performed with all data (outliers included).

An unpaired Mann-Whitney test with a 95% confidence level was performed. Patients vs. controls were compared for each ROI. Exact two-tailed *P*-values are listed in Table 6. The data are visualized with boxplots with added scatter plots and the *P*-value of the Mann-Whitney test in Figure 1. Each dot represents the relative expression ratio of each control or patient obtained with Equation (1). The number of controls and patients used for MSRE-qPCR analysis was lower than the initial 65 controls and 76 patients due to the limited amount of DNA for specific samples and difficulties with qPCR.

## Discussion

PFAPA syndrome is the most common pediatric fever syndrome, and its etiology is unknown. So far, no clear genetic causes have been found. In this study, we analyzed differential methylation patterns in a cohort of 75 PFAPA patients. All patients included in the study fulfilled the clinical criteria for PFAPA syndrome (5). The majority of patients analyzed were boys (58.7%). During the fever episode, pharyngitis and adenitis were the most common symptoms (94.7 and 89.3%, respectively). All three major symptoms (adenitis, pharyngitis, and aphthous stomatitis) were present in 52% of the patients. The included patients had previously been analyzed in an independent study, and genetic analysis was performed for four genes: *AIM2, NLRP3, MEFV*, and *MVK*. No clinically significant variants were found (18).

Relative quantification with the MSRE-qPCR method proved to be a quick and efficient way to estimate differentially methylated DNA regions, which were identified beforehand by MeDIP and MBD. Our results showed that there are two regions, one inside the second intron of the PIK3AP1 gene and a second one inside the fifth intron of SPON2, which have a statistically significant difference in methylation in PFAPA patients when compared to healthy controls.

Both MeDIP and MBD have the advantage of identifying regions with differential methylation over the whole genome, while MSRE-qPCR is more suitable to analyze the few previously identified candidate regions. MeDIP favors regions with low CpG density and MBD favors regions with higher CpG density (29), which was also observed in our case. All three methods are a suitable way to identify differentially methylated genomic regions, though they cannot determine how many and which CpGs are methylated specifically.

The *PIK3AP1* gene was identified as more methylated in PFAPA patients by MeDIP, which the MSRE-qPCR method confirmed, but only for a part of the said region. The MBD results identified a region of *SPON2* as more methylated in PFAPA patients, while MSRE-qPCR later revealed that this is not the case for the whole identified region. The first half had lower methylation levels in PFAPA patients, while the second half had higher methylation levels. A possible reason for such divergence with the results of the first half of *SPON2* could be the specifics of the process of broad peak identification by the MACS2 algorithm, where a wider area is incorporated into the final signal peak identification, partially influenced by the final NGS library insert size as well. Nevertheless, there is a measurable difference in the methylation pattern in the fifth intron region of the *SPON2* gene.

The B-cell adapter protein (BCAP, *PIK3AP1* gene) is a phosphoinositide 3-kinase (PI3K) binding protein (30) and an important inhibitor of proliferation and myeloid cell differentiation that works in a cell-intrinsic manner (31). The PI3K signaling cascade influences cell proliferation and survival, metabolic reprogramming, and cellular migration. As a PI3K adaptor, BCAP is a key regulator of PI3K signaling and T-cell development into effector and memory cells (32). It is expressed in lymphoid as well as in myeloid cell populations (31–34). BCAP regulates inflammatory response. BCAP deficiency results in exaggerated innate immune response, leading to higher CD4^+^ activation (35) and to more proliferative cells (31). As a macrophage signaling adaptor protein, it can dampen NLRP3 and NLRC4 inflammasome activation through interaction with the caspase-1 inhibitor (36). Altogether, BCAP could play a part in influencing systemic inflammation, which PFAPA syndrome is known for.

Spondin-2 (also called mindin, *SPON2* gene) is an extracellular matrix protein that functions as a pattern recognition molecule (PRM) for initiating innate immune responses, as well as an integrin ligand for inflammatory cell recruitment and T-cell priming (37–39). It has been shown that, *in vivo*, it is crucial for the efficient clearance of bacterial (37) and viral (40) infections. Spondin-2/mindin-induced signaling could be as important as other, better-defined signaling pathways, such as TLR signaling. He et al. have proposed that mindin-mediated carbohydrate recognition of microbial pathogens represents a secondary stimulation essential for activation of innate immune cells (37). Additionally, it has been suggested that it has a role in the immune response against tumor cell growth and migration (41). Little else is known of spondin-2 regarding immune regulation; however, as an extracellular PRM, it could have a role in inflammation, since extracellular PRMs are able to complement activation, opsonization, agglutination, neutralization, and regulation of inflammation (42).

Epigenetic modifications, including DNA methylation, can affect gene expression and, consequently, can be used as a disease biomarker (43). Methylation of CpG dinucleotides is one of the principal epigenetic mechanisms (44). Regions rich in CpGs are called CpG islands (CGI). Unmethylated promotor CGIs are generally associated with transcriptionally active genes, whereas hypermethylated promotor CGIs result in gene transcription repression. Furthermore, methylation levels inside the gene correlate with gene expression, especially the first intron methylation (45). The majority of research on disease-related DNA methylation has been done in the field of cancer (43); however, there is increasing evidence that epigenetic dysregulation plays a role in autoinflammatory diseases as well (46).

The role of DNA methylation of intragenic regions is less clear, though evidence exists that methylated intragenic regions influence gene transcription (47). Partial methylation of the coding region can inhibit gene expression (48, 49), and even a few methylated cytosines can inhibit a flanking promoter, but a threshold of modified sites is required (50). Elevated DNA methylation in intragenic regions usually correlates with silencing of the associated gene (51). Blattler et al. showed that changes in DNA methylation within bodies of genes played a much larger role than changes in promotor regions (52). The methylation level of the first intron is inversely associated with gene expression, and this association is conserved regardless of the species or tissue. Methylation tends to increase with distance from the first exon–first intron boundary, and its effect on gene transcription decreases with downstream distance from the first exon (45, 53). The study of DNA methylation during monocyte differentiation revealed that a high proportion of the changes occurred in non-promoter regulatory regions, mainly enhancers in gene bodies and intergenic regions (54). Methylation patterns in the *MEFV* exon in pediatric patients with Familial Mediterranean Fever correlated with expression of the same gene; the observed slight increase in DNA methylation of the second exon in patients correlated with decreased expression (22).

The observed differential methylation of the second intron in *PIK3AP1* (10:98425953–98426190) could be of great significance regarding changed expression. BCAP functions as a checkpoint to restrict TLR signaling and production of inflammatory cytokines (55). We hypothesize that the higher methylation found in the second intron of *PIK3AP1* in PFAPA patients could lead to lower expression of the BCAP protein and cause a disrupted inhibition of inflammation, leading to exaggerated inflammation or response to environmental stimuli (for example an infection). The cause of this particular differential methylation is unknown, and DNA methylation can be influenced by a range of external factors, such as diet, drugs, and infections (56, 57). There is evidence of infection-induced hypermethylation of *PIK3AP1* promoter and downregulation of its expression (58) and evidence of hypomethylation and increased expression of *PIK3AP1* triggered by low levels of folic acid (59). Infections are proposed contributors to the pathogenesis of PFAPA (15, 60).

The second half of the spondin-2 region (4:1163480–1163727, GRCh37) that was investigated and showed higher methylation in PFAPA patients is also part of a CTCF binding region (4: 1163601–1163800, GRCh37). CTCF is a transcription factor, chromatin organizer, and insulator protein that was initially discovered as a transcriptional repressor; however, its exact mechanisms remain unknown (61). We hypothesize that the observed increased methylation could potentially inhibit CTCF binding. As an insulator protein, CTCF can influence gene transcription by restricting the binding of transcription enhancers. Its binding sites are located far from the transcriptional start sites, and their distribution is strongly correlated with genes (62). However, without gene expression analysis, we cannot confirm whether the changed methylation in *PIK3AP1* and *SPON2* influences their expression and in what way.

DNA was isolated from whole blood, meaning we analyzed methylation in DNA from the whole blood cell population with unknown ratios. Samples were collected during an afebrile period, patients were asymptomatic, and we assumed that blood cell populations were normal and comparable based on the facts that changes in white blood cell counts are associated with febrile episodes (13, 63) and that the concentrations of white blood cells in afebrile PFAPA patients are comparable to those in healthy children (63). Our results indicate that there is a portion of blood cells with differentially methylated DNA in these two genes and that the number of cells or the specific clonal subpopulation of cells was high enough to generate a measurable signal. Currently, there is no clear evidence which cell subpopulation is involved. High monocytes and high neutrophils seem to be the most consistently reported changes in blood cell populations during PFAPA febrile attacks (13, 63, 64) and could be the possible source of the differential DNA methylation we observed.

A limitation of our study, in addition to the mixed cell populations as our source of DNA, is the fact that 15 patients (20%) had previously received methylprednisolone, which could affect their DNA methylation patterns. We do not know if it affected the regions in question or in what way and for how long. Steroids do have an effect on the disease, but since their effect is not permanent, they only stop the episode and not the disease itself (65–67). In order to overcome this limitation, we re-analyzed data excluding patients that received steroid treatment and obtained similar results. Significance did not change, except for the second half of the *SPON2* region, which crossed the significance line but still showed a tendency of hypermethylation. Loss of significance could also be partly attributed to reduced sample size. Additional studies investigating the effect of methylprednisolone should be conducted. Another limitation would be that the test and control groups were not of the same age (4.1 and 5.3 years, respectively). The median age of our control group was intentionally above five, which is when the fevers generally begin to appear (5–7). Altogether, the results from our methods do not inform us of the identity and number of CpGs involved. We also cannot tell in which cells this difference in methylation occurs, since the source of our analyzed DNA was whole blood. For a more detailed look into the possible role of DNA methylation in the pathogenesis of PFAPA, DNA isolated from different cell lineages should be analyzed.

Both the B-cell adapter protein through the PI3K activation pathway and spondin-2 as an extracellular pattern recognition molecule are involved in the primary stages of immune responses. BCAP acts as an inhibitor of receptor signaling, mainly TLR and spondin-2, possibly as a recruiter or activator of T cells, or by working as an opsonin that activates complement. The role of changed DNA methylation in autoinflammation certainly needs further investigation, as does the role of BCAP and spondin-2 in the etiology of PFAPA. Differential methylation of genes *PIK3AP1* and *SPON2* is not reported in association with other similar autoinflammatory diseases; however, *PIK3AP1* was associated with autoimmunity (68), and its increased expression was shown to promote TLR7-driven lupus-like disease (69). Differential expression and methylation of both genes have been linked with cancer. Changed methylation of *PIK3AP1* is associated with neuroblastoma (70), and its upregulation is associated with Waldenström macroglobulinemia (71). Hypomethylation of the promoter of *SPON2* and its increased expression is associated with prostate cancer (72, 73); its upregulation is also associated with colorectal cancer (74).

As far as the clinical impact of these data is concerned, this kind of change in methylation would not be detected by Sanger sequencing, because the proportion of differentially methylated DNA molecules is too small. Therefore, a more advanced quantification method is needed, such as MSRE-qPCR. Nevertheless, the cutoff values for the positive MSRE-qPCR results should be carefully examined, set, and validated.

## Conclusions

Whole-genome methylation screening analysis methods, such as MeDIP and MBD are a useful tool for identifying differentially methylated genomic regions. MSRE-qPCR proved to be a reliable, quick, and cost-effective method of confirming results and showed potential applicability in translation of the research into clinical practice. The changed methylation patterns in PIK3AP1 and SPON2 that we observed in PFAPA patients point to novel and still unknown roles of BCAP and spondin-2 in the etiology of PFAPA. Furthermore, likely transient changes of DNA methylation patterns are potentially a novel direction in research into the molecular mechanisms leading to PFAPA development.

## Data Availability Statement

The datasets generated for this study are available on request to the corresponding author.

## Ethics Statement

The studies involving human participants were reviewed and approved by Ethics Committee of the Republic of Slovenia. Written informed consent to participate in this study was provided by the participants' legal guardian/next of kin.

## Author Contributions

EL, JK, TT, and TR contributed the conception and design of the study and performed the actual experiments and analysis. EL wrote the first draft of the manuscript. JK wrote the sections of the manuscript. NT and DP selected the included patients and collected data and samples. MD and TA contributed equally and were responsible for the final approval of the submitted version. All authors contributed to the article and approved the submitted version.

## Conflict of Interest

The authors declare that the research was conducted in the absence of any commercial or financial relationships that could be construed as a potential conflict of interest.
