# Supplementary material for: PIK3AP1 and SPON2 Genes Are Differentially Methylated in Patients With Periodic Fever, Aphthous Stomatitis, Pharyngitis, and Adenitis (PFAPA) Syndrome
Source: Front Immunol. 2020 Jul 23;11:1322. doi: 10.3389/fimmu.2020.01322 (PMC7390842; doi:10.3389/fimmu.2020.01322)
Supplement: Supplementary file 2 [file Table_2.DOCX]

Supplementary Material

## Supplementary Figures


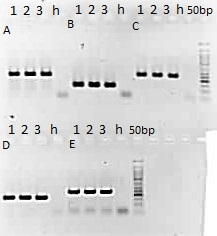


**Supplementary Figure 1.** Agarose gel (2%) electrophoresis of PCR products from primer evaluation (A: Hbb, B: PIK1, C: PIK2, D: SPON1, E: SPON2). For each three reactions with three different samples (1-3) and one sham reaction (h) with water. 50bp DNA ladder was used as size marker (fragments of 50, 100, 150, **200**, 250, 300, 350, 400, 450, **500**, 550, 600, 650, 700, 766, 916 and 1350 bp length).


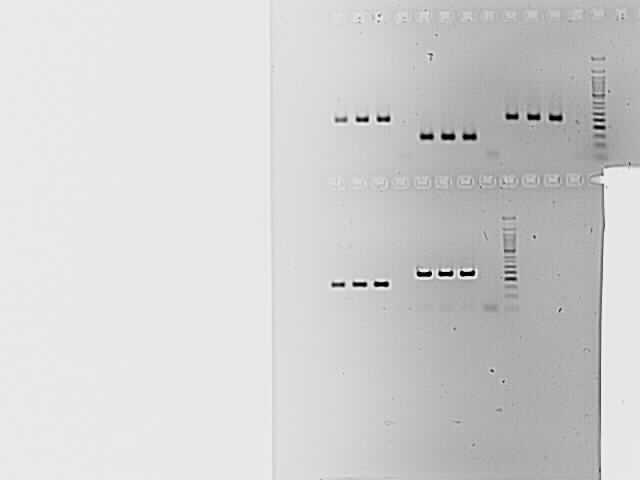


**Supplementary Figure 2:** Unedited and uncropped image of Supplementary Figure 1.
